# An engineered arginine-rich α-helical antimicrobial peptide exhibits broad-spectrum bactericidal activity against pathogenic bacteria and reduces bacterial infections in mice

**DOI:** 10.1038/s41598-018-32981-3

**Published:** 2018-10-02

**Authors:** Chin-Hao Yang, Yi-Cheng Chen, Shih-Yi Peng, Andy Po-Yi Tsai, Tony Jer-Fu Lee, Jui-Hung Yen, Je-Wen Liou

**Affiliations:** 1 Institute of Medical Sciences, Tzu Chi University, Hualien, Taiwan; 2 Department of Medicine, Mackay Medical College, New Taipei, Taiwan; 3 Department of Biochemistry, School of Medicine, Tzu Chi University, Hualien, Taiwan; 4 Ph.D. Program in Translational Medicine, Tzu Chi University/Academia Sinica, Taipei, Taiwan; 5 Department of Medical Research, Buddhist Tzu Chi General Hospital, Hualien, Taiwan; 6 Department of Pharmacology, Southern Illinois University School of Medicine, Springfield, IL USA; 7 Department of Molecular Biology and Human Genetics, Tzu Chi University, Hualien, Taiwan

## Abstract

The increase in the prevalence of antibiotic-resistant bacteria has become a major public health concern. Antimicrobial peptides (AMPs) are emerging as promising candidates addressing this issue. In this study, we designed several AMPs by increasing α-helical contents and positive charges and optimizing hydrophobicity and amphipathicity in the Sushi 1 peptide from horseshoe crabs. A neural network–based bioinformatic prediction tool was used for the first stage evaluations of peptide properties. Among the peptides designed, Sushi-replacement peptide (SRP)-2, an arginine-rich and highly α-helical peptide, showed broad-spectrum bactericidal activity against both Gram-positive and Gram-negative bacteria, including methicillin-resistant *Staphylococcus aureus* and multidrug-resistant *Acinetobacter baumannii*; nevertheless, it showed little hemolytic and cytotoxic activity against mammalian cells. Atomic force microscopy results indicated that SRP-2 should interact directly with cell membrane components, resulting in bacterial cell death. SRP-2 also neutralized LPS-induced macrophage activation. Moreover, in an intraperitoneal multidrug-resistant *A*. *baumannii* infection mouse model, SRP-2 successfully reduced the bacterial number in ascitic fluid and tumor necrosis factor-α production. Our study findings demonstrate that bioinformatic calculations can be powerful tools to help design potent AMPs and that arginine is superior to lysine for providing positive charges for AMPs to exhibit better bactericidal activity and selectivity against bacterial cells.

## Introduction

Since the discovery of the first antibiotic, penicillin, in 1928^1^, antibiotics have become one of the closest allies of mankind in combating bacterial infections. However, after a long history of antibiotic use, bacteria have evolved means to resist most of the common antibiotics. Searching or developing new strategies for the treatment of bacterial infections has thus become a vital task. Antimicrobial peptides (AMPs) have been proposed as a promising class of antibiotics for this purpose^2–4^. AMPs are important components of the innate and adaptive immune systems to protect organisms from microbial infections^5,6^. Moreover, AMPs have been isolated from single-celled microorganisms, plants, invertebrates, amphibians, birds, fish, and mammals, including humans^7–10^. Some AMPs have been developed into commercial drugs^11,12^ or suggested as candidates for new antimicrobial agents^13^. Nevertheless, because most novel peptide drug developments fail between their identification and introduction to the market^14^, methodologies to effectively generate lead drugs for further tests should be developed. Accumulated information from numerous studies on the characteristics of AMPs has provided clues for further AMP development. Although AMPs can adopt different conformational structures, including β-sheet and random-coil structures, most of them have α-helical structures^15,16^. AMPs also exhibit a certain degree of generalized properties, including amphipathicity, mean hydrophobicity, and net cationic charge^8,17–26^. Although peptide amphipathicity is an important factor responsible for the interaction of peptides with amphipathic biological membranes, the net cationic nature of AMPs is suggested to be responsible for peptide selectivity^16,18,27^. Positively charged AMPs interact strongly with negatively charged bacterial membranes that contain a large proportion of negatively charged molecules, including negatively charged lipids such as phosphatidylglycerol and cardiolipin^16^, lipoteichoic acids in the peptidoglycan for Gram-positive bacteria, and lipopolysaccharides (LPSs) in the outer membrane for Gram-negative bacteria^28^. Because the outer leaflet of mammalian membranes contains mostly neutrally charged lipids, including sphingomyelin, phosphatidylcholine, and sterols^16^, the architectural differences between mammalian and bacterial membranes should provide a feasible means for cationic AMPs to selectively target bacteria.

In this study, we used the sequence of a 34-amino-acid AMP named Sushi 1 (S1) obtained from horseshoe crab hemocytes^29^ as a template, and we made substitutions of amino acids in the sequence to generate peptides for more powerful and broad-spectrum bactericidal applications and higher bacterial/mammalian cell selectivity. As mentioned, cationic charges, amphipathicity, and helical structures are important for AMP functions; we thus changed amino acids in the sequence to increase the α-helix content in the peptide structures and increase the positive charge levels and amphipathicity of the peptides. The derived peptides were then tested for their bactericidal activities and hemolysis effects. The most effective peptide was selected for further biophysical examinations including circular dichroism (CD) and atomic force microscopy (AFM), as well as *in vitro* and *in vivo* tests of the peptides on the bacteria-related response in cells and mice. The aim of this study was to develop a methodology for the design of more effective AMPs and to evaluate the structure–function relationships of the peptides.

## Materials and Methods

### Bioinformatic design of AMPs

The sequence-dependent peptide characteristics, including amphipathicity, mean hydrophobicity, helicity, and net charge, were calculated. The helicities of the peptides were calculated using the hierarchical neural network secondary structure prediction method available on the NSP@ server http://npsa-pbil.ibcp.fr/ at the following https://npsa-prabi.ibcp.fr/cgi-bin/npsa_automat.pl?page=/NPSA/npsa_hnn.html. The mean hydrophobicities and amphipathicities of the peptides were calculated through the analysis procedure on the HeliQuest service at http://heliquest.ipmc.cnrs.fr/. The mean hydrophobicity <H> of a peptide was calculated as follows^30^:

1 $$ < \,H > =\frac{1}{N}{\sum }_{(n-1)}^{N}{H}_{n}$$

where N is number of amino acids in the sequence and *H*_*n*_ is the hydrophobicity of the n^th^ amino acid in the sequence. The hydrophobicity of each amino acid was indicated in a previous study^31^.

The mean amphipathic moment <*µ*H> of a peptide was calculated as follows^30^:

2 $$ < \,\mu H > =\frac{1}{{\rm{N}}}{[{[{\sum }_{{\rm{N}}-1}^{{\rm{N}}}{{H}}_{{\rm{n}}}\sin (n\delta )]}^{2}+{[{\sum }_{{n}-1}^{{N}}{{H}}_{{n}}\cos ({n}\delta )]}^{2}]}^{\frac{1}{2}}$$

where nδ is the angle separating side chains along the backbone, with δ = 100° for an α-helix^30^.

Distributions of amino acids and amphipathicity assessments for α-helical peptides were projected in helical wheel diagrams, which were also created from the web server HeliQuest.

The foundation template for the peptide designs was the S1 peptide derived from the hemocyte factor C protein of horseshoe crab^32^. The wild-type S1 peptide was previously measured to be a largely unstructured peptide in aqueous environments^28^, although its α-helical structure has been suggesged to be induced when it interacts with bacterial components such as membrane lipids^28^ and LPS^18,32,33^, or structure-promoting exogenous elements such as 50% trifluoroethanol^32^. In this study, we substituted amino acids in various positions in the S1 sequence, in an attempt to increase the α-helix content and the net positive charge of the peptides. Three mutant peptides, Sushi-replacement peptides (SRPs) 1, 2, and 3, were derived and synthesized for further tests.

### Peptide synthesis and purification

The designed peptides used in this study were synthesized using a standard 9-fluorenyl methoxy carbonyl (F-moc) solid-phase synthesis technique on an automatic peptide synthesizer (433A, Applied Biosystems, MA, USA). The synthesized peptides were purified using a reverse-phase high-performance liquid chromatography (HPLC) instrument (Waters 600, MA, USA) equipped with a preparative reverse-phase column (XBridge BEH 130 Prep C18 Column, 10 μm OBD 19 × 250 mm, Waters, MA, USA). The purities of purified peptides were over 95% as confirmed with HPLC and ESI mass spectrometry (examples of the purity checks are given in Supplementary Fig. S1).

### Bacterial strains

*Escherichia coli* (ATCC 25922) and *Staphylococcus aureus* (ATCC 25923) were bacterial strains from the American Type Culture Collection (ATCC). Methicillin-resistant *S*. *aureus* (MRSA, BCRC 80277) was obtained from the Bioresource Collection and Research Center (BCRC), Hsinchu, Taiwan. Moreover, multidrug-resistant *Acinetobacter baumannii* (MDRAB) was a clinical isolate from Buddhist Tzu Chi Medical Center, Hualien, Taiwan.

### Bactericidal assay

The antimicrobial activities of the wild-type Sushi peptide, SRP-1, SRP-2, and SRP-3 were determined by bactericidal assays on laboratory strains of *E*. *coli*, *S*. *aureus*, MRSA, and MDRAB. For peptide treatments, the bacteria were grown to the mid-logarithmic phase in Luria broth. The bacterial cells were collected by centrifugation at 5000 rpm and washed twice with phosphate-buffered saline (PBS, pH 7.4). To assay the bactericidal activities of the peptides, the peptides were added to PBS containing inoculants of the test bacteria (5 × 10^5^ CFU/mL) to final concentrations of 50, 25, 12.5, and 6.25 μM. The bacterial samples were treated with the peptides for 2 h at 37 °C. The bacterial suspensions were then diluted and spread on LB- agar plates. After overnight incubation at 37 °C, bacterial colonies were counted. Bacterial survival rate was determined as follows:

3 $$\begin{array}{rcl} \% \,{\rm{survival}}\,{\rm{rate}} & = & [({\rm{Colonies}}\,{\rm{formed}}\,{\rm{from}}\,{\rm{peptide}} \mbox{-} {\rm{treated}}\,{\rm{sample}})\\  &  & /({\rm{Colonies}}\,{\rm{formed}}\,{\rm{from}}\,{\rm{negative}}\,{\rm{control}})]\times 100 \% \end{array}$$

### Peptide hemolysis assays

For examining the hemolysis activities of the peptides, whole blood was used in the tests. Whole blood suspensions of 400 μL containing erythrocyte cells were mixed with the S1 wild-type and mutant peptides to a final peptide concentration of 50 μM. The suspensions were incubated at 37 °C on a rotary shaker for 35 min. The samples were centrifuged at 2000 rpm for 10 min. Hemoglobin release was monitored by measuring the absorbance of the supernatant at 540 nm using a microplate ELISA reader (BioTek, Winooski, VT, USA). The blood samples treated with 1% Triton X-100 were used as positive controls and were determined to have 100% hemolysis. The percentages of hemolysis induced by peptides were calculated as follows:

4 $$[({{\rm{A}}}_{{\rm{p}}}-{{\rm{A}}}_{{\rm{c}}})/({{\rm{A}}}_{{\rm{T}}}-{{\rm{A}}}_{{\rm{c}}})]\times 100 \% $$

where A_p_ is the absorbance of a peptide-treated sample; A_c_ is the absorbance of an untreated control sample; and A_T_ is the absorbance of a Triton X-100–treated sample.

### Cytotoxicity assay on mammalian cells

For measuring the cytotoxicity of the peptides, a colorimetric 3-bis-(2-methoxy-4-nitro-5-sulfophenyl)-2H-tetrazolium-5-carboxanilide (XTT) dye reduction assay was employed. In the measurements, HMEC-1 cells were used to represent primary human dermal endothelial cells^34^. The experimental procedure followed previously described methods^35^. In short, HMEC-1 cells were plated onto 96-well plates at 2 × 10^4^ cells/100 μL/well in MCDB 131 media and 10% fetal calf serum, and they were then incubated under a fully humidified atmosphere of 95% air and 5% CO_2_ at 37 °C overnight. The peptide was then added to the cell cultures at final concentrations of 0.78–25 μM. After 2 h of treatment, 50-μL freshly prepared XTT reagent was added to the cell cultures, and the cells were further incubated for 12 h at 37 °C. For each sample, the absorbance at 450 nm was then measured using a microplate ELISA reader (BioTek, Winooski, VT, USA).

### Atomic force microscopy

To visualize the peptide bactericidal effects on bacterial cells, AFM was performed. In AFM experiments, the bacterial cells were washed twice with PBS and treated with peptides. After the peptide treatments, 10 mL of the bacterial suspension was placed onto a freshly cleaved mica disc (Ted Pella, Redding, CA, USA), and samples were allowed to be adsorbed for 20–40 min. The mica discs were then gently rinsed twice with Milli Q water and dried in a desiccator. The prepared samples were imaged using an AFM instrument (Nanowizard, JPK Instruments, Germany) operating in contact mode. Images were collected at a resolution of 512 × 512 pixels using a scanning speed of 1.0–2.0 Hz. The AFM probes used were oxide-sharpened silicon nitride (Si_3_N_4_) probes (OMCL-TR400PB-1, Olympus, Japan) with a spring constant of 0.02 N/m. The SPM image processing package version 3.1.6 (JPK Instruments) was used for AFM image data processing and analysis.

### CD spectroscopy

The secondary structures of peptides in different environments were measured using CD spectroscopy. The CD spectra were collected at 25 °C on a CD Spectrometer Model 410 (AVIV, Biomedical, USA) equipped with a 0.1-mm path-length quartz cell. The final concentration of SRP-2 was 15 μM. Peptide samples in PBS (pH 7.4) were under the conditions of 30–240 mM sodium dodecyl sulfate (SDS) micelles, 50% trifluoroethanol (TFE), or 6–48 μM *E*. *coli* O127:B8 LPS (Sigma-Aldrich, USA). The spectra were recorded at 200–250 nm using a scan speed of 10 nm/min. At least eight scans were collected and averaged for each sample. CD measurements on the interactions of SRP-2 with actual bacterial cells were also carried out, using the method described by Avitabile *et al*.^36^ with modifications. In this experiment, SRP-2 at a final concentration of 15 μM was interacted with MDRAB at different cell concentrations (2.5, 5, 10 × 10^5^ CFU/mL) in PBS (pH 7.4). The CD spectra of the peptide-bacteria mixtures at different interaction time (0, 1, 2, and 3 h) were recorded with a 1-mm path-length quartz cell at 25 °C. The CD spectra–based secondary structure analysis of the peptides was performed using the web server DichroWeb^37^. The K2D algorithm, an unsupervised learning neural network, was used to estimate the related secondary structures of the peptides^38^.

### Infections and treatments on mice

Animal experiments were conducted in accordance with the regulations set out in the Animals (Scientific Procedures) Act 1986 and performed as approved by the Tzu Chi University Institutional Animal Care and Use Committee (No. 102068). Male BALB/c mice (aged 8–10 weeks) were purchased from the Animal Center, National Applied Research Laboratories (Taipei, Taiwan). The mice were housed in a temperature-controlled, light-cycled facility in accordance with the regulation of the National Institutes of Health Guide for the Care and Use of Laboratory Animals (DHHS publication No. NIH 85–23, revised 1996). The bacteria applied to infect the mice were log-phase MDRAB, appropriately diluted by PBS to yield the final bacterial concentration of 1 × 10^7^ CFU/mL. Animals were infected by intraperitoneal (i.p.) injection with 0.2 mL of the bacterial suspensions. Four mice per group received a 0.2-mL i.p. injection of normal saline (control group) or SRP-2 (5 mg/kg) approximately 5 min after the bacterial challenge. The mice were monitored for 4 h and then euthanized by cervical dislocation. PBS (5 mL) was injected into the peritoneal cavity; subsequently, ascitic fluid was obtained by aspirating peritoneal lavage. Moreover, 0.1 mL of blood was withdrawn by cardiac puncture. The obtained samples were directly plated. MDRAB burden in blood and ascites was assessed by colony counts after growth on agar at 37 °C overnight. Serum and ascites of the MDRAB-infected mice were also collected after the treatments for the measurements of tumor necrosis factor-α (TNF-α) production. TNF-α in mouse serum and ascites was measured using a commercial TNF-α ELISA kit (Invitrogen, Carlsbad, CA, USA). To evaluate the ability of SRP-2 to prevent lethal bacteremia, mice were challenged by i.p. injection of MDRAB (2 × 10^8^ CFU/mouse, 200 μL/mouse). At 1 h after the bacterial injection, the mice were i.p. injected with 10 mg/kg mouse of SRP-2 (100 μL/mouse, n = 8), or PBS (100 μL/mouse, negative control, n = 7). Death of the mice were recorded every 6 h and survival was tracked for 5 days.

### Nitric oxide production

For nitric oxide (NO) production measurements, RAW 264.7 macrophage cells were cultured and maintained in Dulbecco’s modified Eagle’s medium (HyClone, Logan, UT, USA) containing antibiotics (100 units/mL of penicillin G and 100 units/mL of streptomycin) and heat-inactivated 10% fetal bovine serum (Invitrogen) at 37 °C in an incubator under 5% CO_2_. RAW 264.7 cells were subcultured in a 24-well plate for experiments. These cells at 2 × 10^5^ cells/well were cotreated with 100 ng/mL of LPS (*E*. *coli* O127:B8, Sigma-Aldrich, St. Louis, Missouri, USA) and 0.3–10 μM SRP-2 for 24 h. The NO production of the LPS-treated cells was indicated by the nitrite concentrations in the culture medium, measured using the Griess reaction^39^. For the reactions, equal volumes of Griess reagent (1% sulfanilamide/0.1% N-(1-naphthyl)-ethylenediaminedihydrochloride/2.5% H_3_PO_4_; Sigma-Aldrich) and culture medium were mixed. After 5 min of incubation, absorbance at 550 nm was measured using a microplate reader (Molecular Devices, Sunnyvale, CA, USA). Sodium nitrite was used as the standard for the calculation of the nitrite concentration in the culture medium.

## Results

### Peptide biophysical characteristic analysis and peptide design

**Figure 1:**
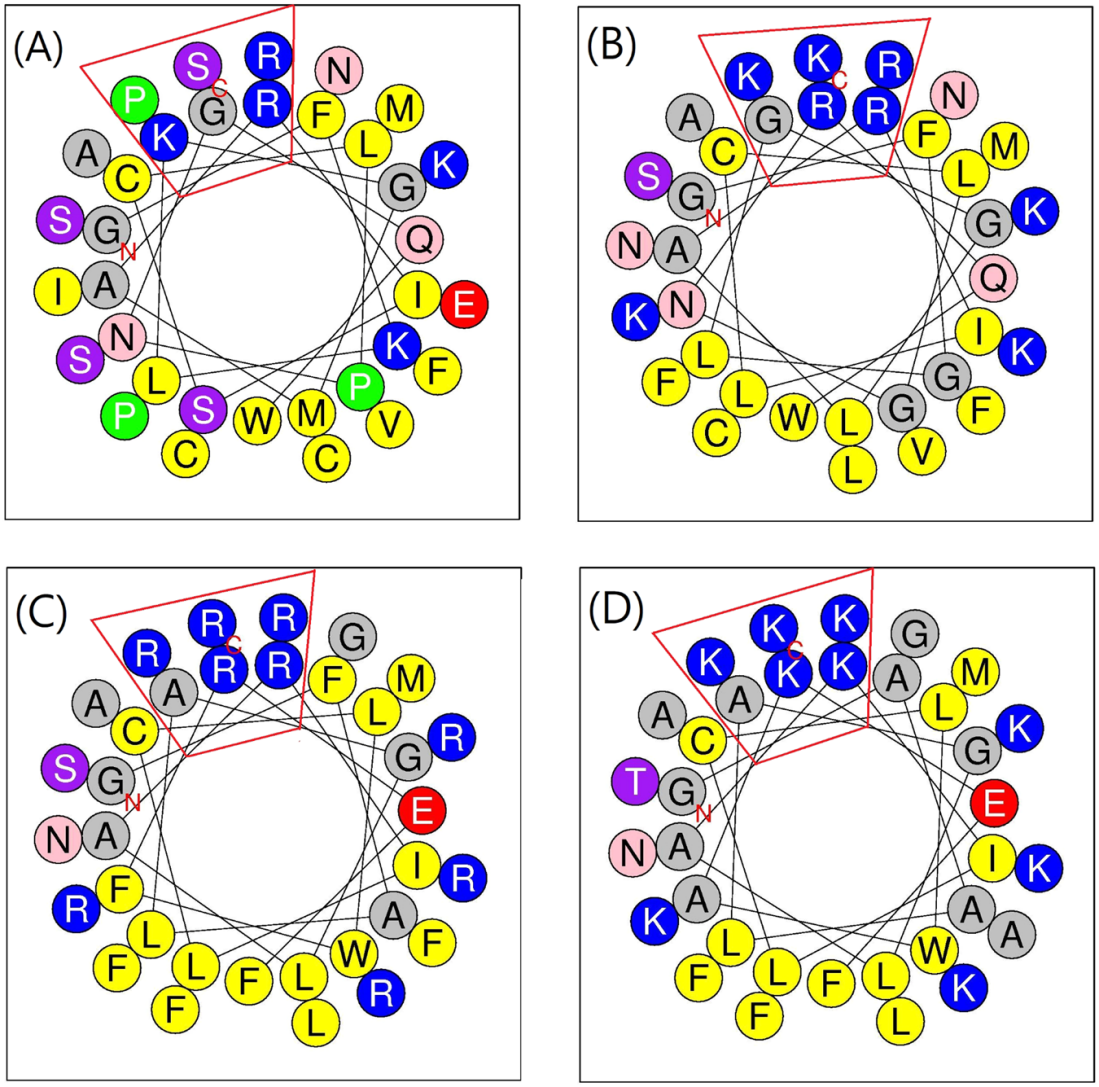
Helical wheel projections of peptides. Helical wheel projections of (**A**) wild-type S1 peptide, (**B**) SRP-1, (**C**) SRP-2, and (**D**) SRP-3. The letters N and C in the projections indicate the N- and C-terminals, respectively. Amino acids in blue color are positively charged, and amino acids in yellow color are hydrophobic.

For the design of AMPs, an S1 peptide from factor C of horseshoe crab hemocytes was applied as the template for peptide engineering. We made amino acid substitutions in the S1 sequence to change the properties of the peptides in order to increase the functional ability of the peptides. Three mutant (amino acid replaced) peptides—namely SRP-1, SRP-2, and SRP-3—were designed. The sequences and the predicted secondary structures of the wild-type and mutant peptides are shown in Table 1, and their calculated biophysical properties are presented in Supplementary Table S1. In a previous study, the wild-type S1 peptide was experimentally suggested to be a random-coiled peptide in aqueous solution^28^. In our predictions, in water environments, the wild-type S1 peptide indeed exhibited a largely random-coil structure (64.71%). However, a considerable amount of extended strands (35.29%) in the structure was also calculated. For the mutant peptides, the amino acid substitutions increased the calculated α-helical structures; the contents of α-helical structures were predicted to be 76.47%, 88.24%, and 85.29% for SRP-1, SRP-2, and SRP-3, respectively. The biophysical characteristic calculations indicated that the amphipathicity, net charges, and mean hydrophobicity of the S1 wild type were 0.258 μH, +4, and 0.491 H, respectively; those of SRP-1 were 0.402 μH, +8, and 0.413 H, respectively; those of SRP-2 were 0.403 μH, +8, and 0.498 H, respectively; and those of SRP-3 were 0.395 μH, +8, and 0.381 H, respectively. To visualize the distribution of amino acids in the peptide helical structures, the amino acids of the peptides were plotted into helical wheel projections (Fig. 1). All the peptides showed amphipathicity when they adopted helical structures (Fig. 1). Furthermore, the distributions of the hydrophobic and hydrophilic amino acids of the mutant peptides in the projections were similar. The major difference among the mutant peptides is the positively charged amino acids used. To provide positive charges of the peptides, in SRP-1, both lysine and arginine were used; in SRP-2, only arginine was used; and in SRP-3, only lysine was used.

**Table 1:** Sequences and predicted secondary structures of wild-type and designed peptides.

Peptides	Size(Da)	Sequences and secondary structure predictions
Wild type	3756.4	GFKLKGMARISCLPNGQWSNFPPKCIRECAMVSS cceeeceeeeeeccccccccccccccecceeccc
SRP-1	3854.6	GFGLGGLARILCLGNRQWSNFFKKLNRKCAMVKK ccchhhhhhhhhhcchhhhhhhhhhhhhhhhccc
SRP-2	4191.0	GFALAGLARILCLWFREFSGFFRRLNRRFAMRRR cchhhhhhhhhhhhhhhhhhhhhhhhhhhhhhcc
SRP-3	3724.6	GAALAGLAKILCLWAKEFTGAFKKLNKKFAMKKK cchhhhhhhhhhhhhhhhhhhhhhhhhhhhhccc

Random-coil, extended-strand, and α-helical structures are indicated as letters c, e, and h, respectively.

### Bactericidal activities of peptides

**Figure 2:**
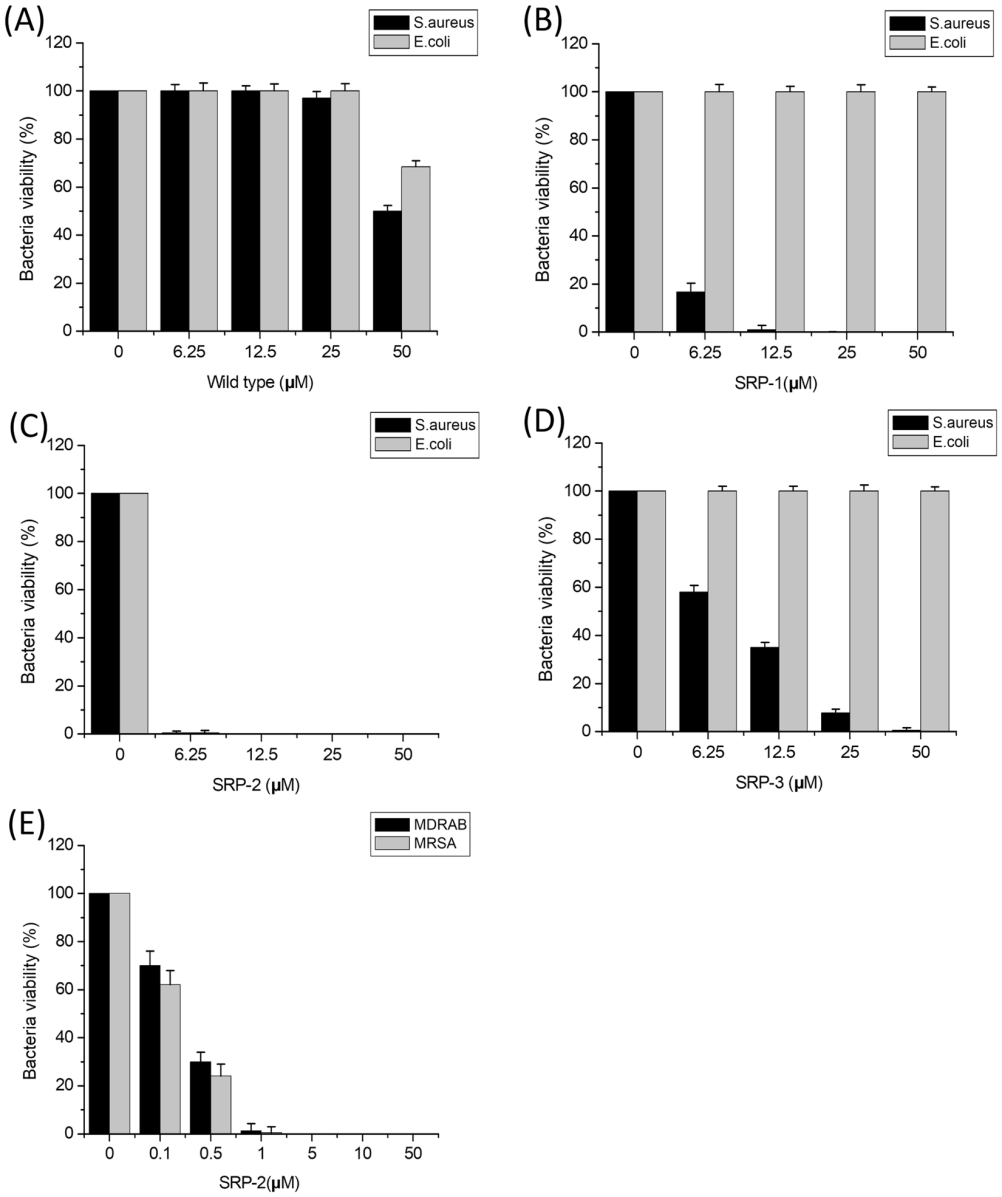
Bactericidal activity of S1 wild type and mutant peptides. (**A**) Bactericidal activity of S1 wild type against *S*. *aureus* and *E*. *coli*. (**B**) Bactericidal activity of SRP-1 against *S*. *aureus* and *E*. *coli*. (**C**) Bactericidal activity of SRP-2 against *S*. *aureus* and *E*. *coli*. (**D**) Bactericidal activity of SRP-3 against *S*. *aureus* and *E*. *coli*. (**E**) Bactericidal activity of SRP-2 against pathogenic MRSA and MDRAB. Bacteria (5 × 10^5^ CFU/mL) were treated with peptides at different concentrations at 37 °C for 2 h. Error bars are standard deviations.

All the wild-type and designed peptides were initially tested for their bactericidal activities against the Gram-positive *S*. *aureus* and Gram-negative *E*. *coli* (Fig. 2). As shown in Fig. 2, the wild-type S1 peptide reduced the number of both bacteria at 50 μM. In the mutant peptides, both SRP-1 and SRP-3 showed greatly improved activity against Gram-positive bacteria, and they were effective at a concentration as low as 6.25 μM. Although SRP-3 showed clear dose-dependent activities against Gram-positive bacteria, SRP-1 seemed to be more effective than SRP-3. However, both SRP-1 and SRP-3 seemed unable to kill Gram-negative bacteria. Among the mutant peptides designed, SRP-2 was the most effective: it was efficient against both Gram-positive and -negative bacteria at 6.25 μM. Therefore, we focused on SRP-2 and further tested its bactericidal effects on the drug-resistant pathogenic bacterial strains MRSA and MDRAB, which were used to represent drug-resistant Gram-positive and Gram-negative bacteria, respectively. SRP-2 was very effective against both bacteria (Fig. 2E). A significant reduction of the bacterial number was observed, even at a peptide concentration of 0.1 μM. Peptide concentrations above 1 μM resulted in total bacterial elimination. The converted minimum bactericidal concentrations (MBCs) of the peptides from our bactericidal experiments are summarized in Supplementary Table S2.

### Cytotoxic activity of SRP-2

**Figure 3:**
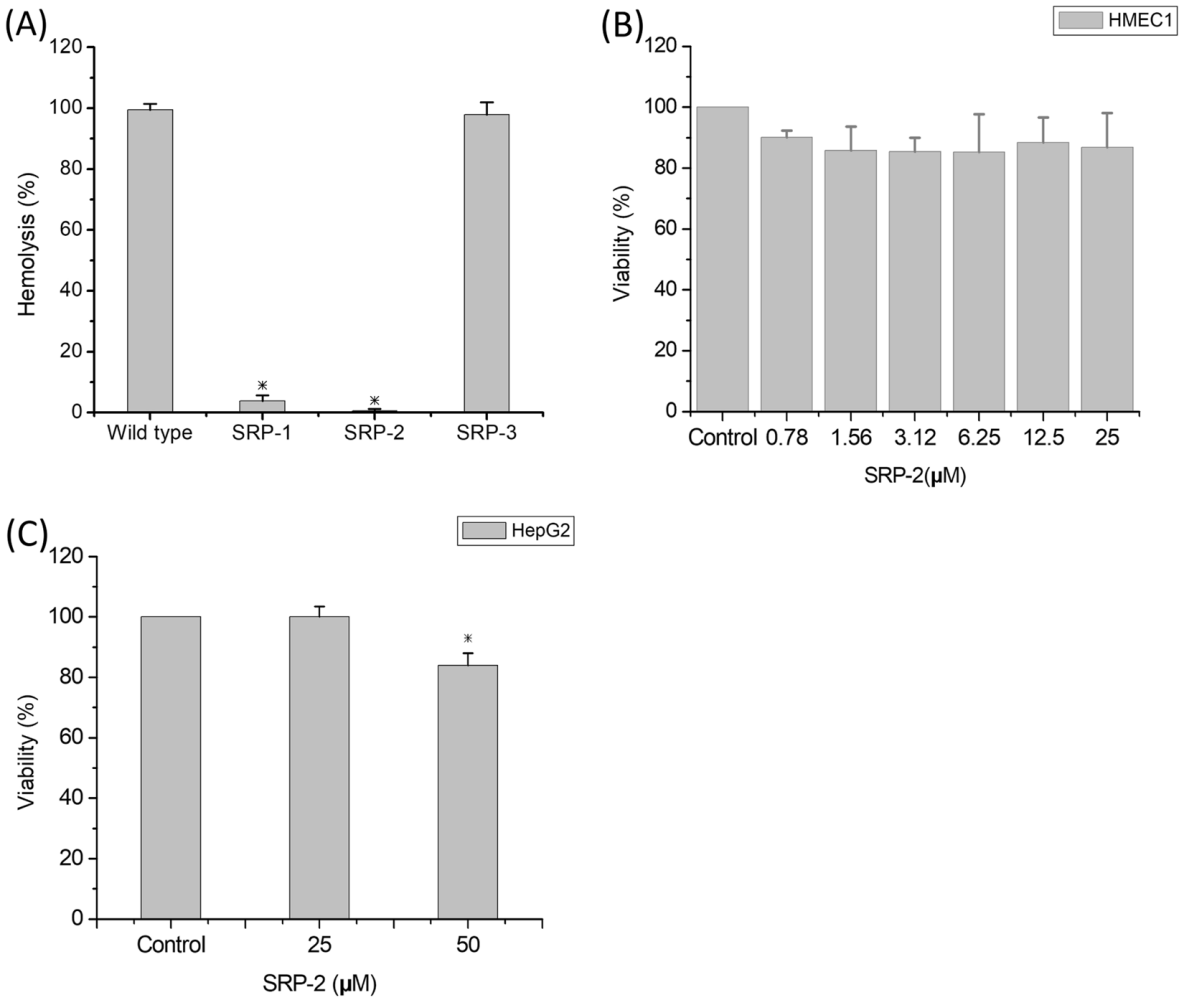
Hemolytic ability and cytotoxicity of peptides. (**A**) Hemolysis effects measured using erythrocytes treated with peptides (50 µM) for 35 min at 37 °C. Erythrocytes treated with 1% Triton X-100 were used as positive controls and set as 100% hemolysis. *p < 0.05, compared to the wild type group. (**B**) HMEC1 treated with SRP-2 at different concentrations. (**C**) HepG2 cells treated with SRP-2 at 25 and 50 μM. For cytotoxicity tests, untreated cells served as controls. *p < 0.05, compared to the control group. Values in the figure are mean ± SD.

To investigate the safety of peptide usage, peptide-induced hemolysis and cytotoxicity against mammalian cells were analyzed *in vitro*. The results are illustrated in Fig. 3. As indicated in Fig. 3A, SRP-1 and SRP-2 showed almost no hemolytic activity at 50 μM, whereas SRP-3 showed strong hemolytic activity at the same concentration. Because SRP-2 was the most effective peptide in this study, we also tested its mammalian cytotoxicity to determine whether it is harmful to mammalian cells. The mammalian cells used in this study were HMEC-1 cells, which have been characterized to retain many of the characteristics of endothelial cells^40^. Because they are a renewable source of human endothelial microvascular cells, they can be used as a replacement for primary human dermal endothelial cells for research studies^41^. The results of cytotoxicity tests of SRP-2 at a peptide concentration of 50 μM are presented in Fig. 3B. SRP-2 was not toxic to mammalian cells at bactericidal concentrations (Fig. 3B). Because AMPs are emerging to be a possible choice in anticancer drug developments^42,43^, we also tested the anticancer ability of SRP-2 against the hepatocellular carcinoma cell line HepG2. A significant reduction (20% reduction) in cell viability was observed at 50 μM (Fig. 3C).

### AFM of SRP-2-treated bacterial cells

**Figure 4:**
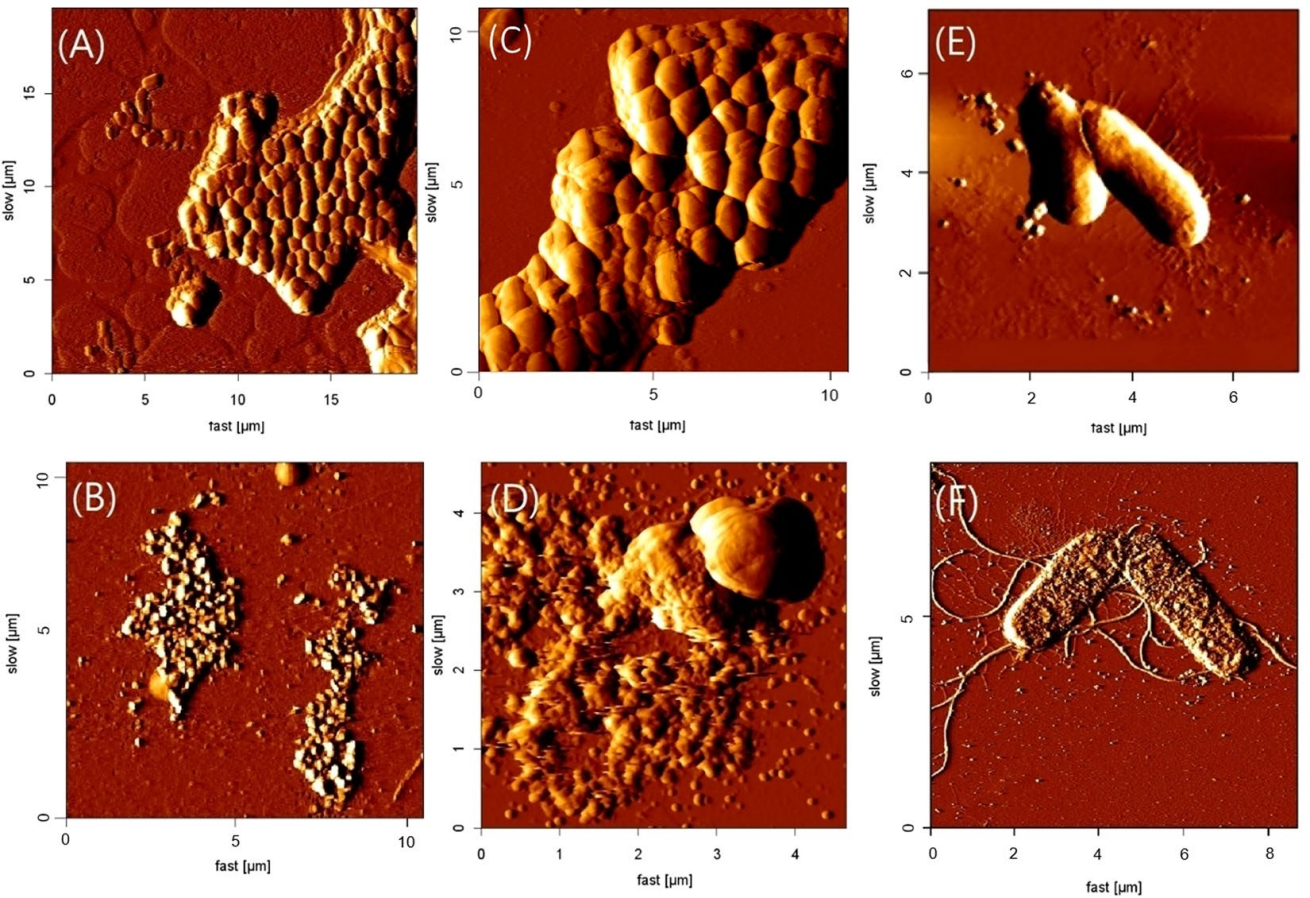
AFM of SRP-2-treated bacterial cells. (**A**,**C** and **E**) present AFM images of untreated MDRAB, MRSA, and *E*. *coli* cells, respectively. (**B**,**D** and **F**) present AFM images of 5 µM SRP-2-treated MDRAB, MRSA, and *E*. *coli* cells, respectively.

We used AFM to visualize the effect of the AMP on bacterial cells. The AFM images of untreated and SRP-2-treated bacterial cells, including MDRAB, MRSA, and *E*. *coli*, are depicted in Fig. 4. The untreated MDRAB cells were observed to be rod shaped and connected (Fig. 4A). After SRP-2 treatment, the connected MDRAB cells were severely damaged and possibly dissolved (Fig. 4B). Similar situations were also observed in the case of MRSA. Typical connected coccus-shaped cells of MRSA were observed in the untreated samples (Fig. 4C), and on treatment with SRP-2, the cells appeared to be disrupted and dissolved (Fig. 4D). We also tested the effect on *E*. *coli*. Figure 4E presents two untreated bacterial *E*. *coli* cells, and Fig. 4F presents two peptide-treated bacterial cells. According to the images of the peptide-treated bacterial cells, we suggest that the bactericidal effects of SRP-2 are related to direct membrane disruptions of the bacterial cells. The disruptions of the bacterial cells were further validated by the increase in membrane permeability of SRP-2 treated MDRAB cells using propidium iodide staining and flow cytometry (Supplementary Fig. S2).

### CD analysis

**Figure 5:**
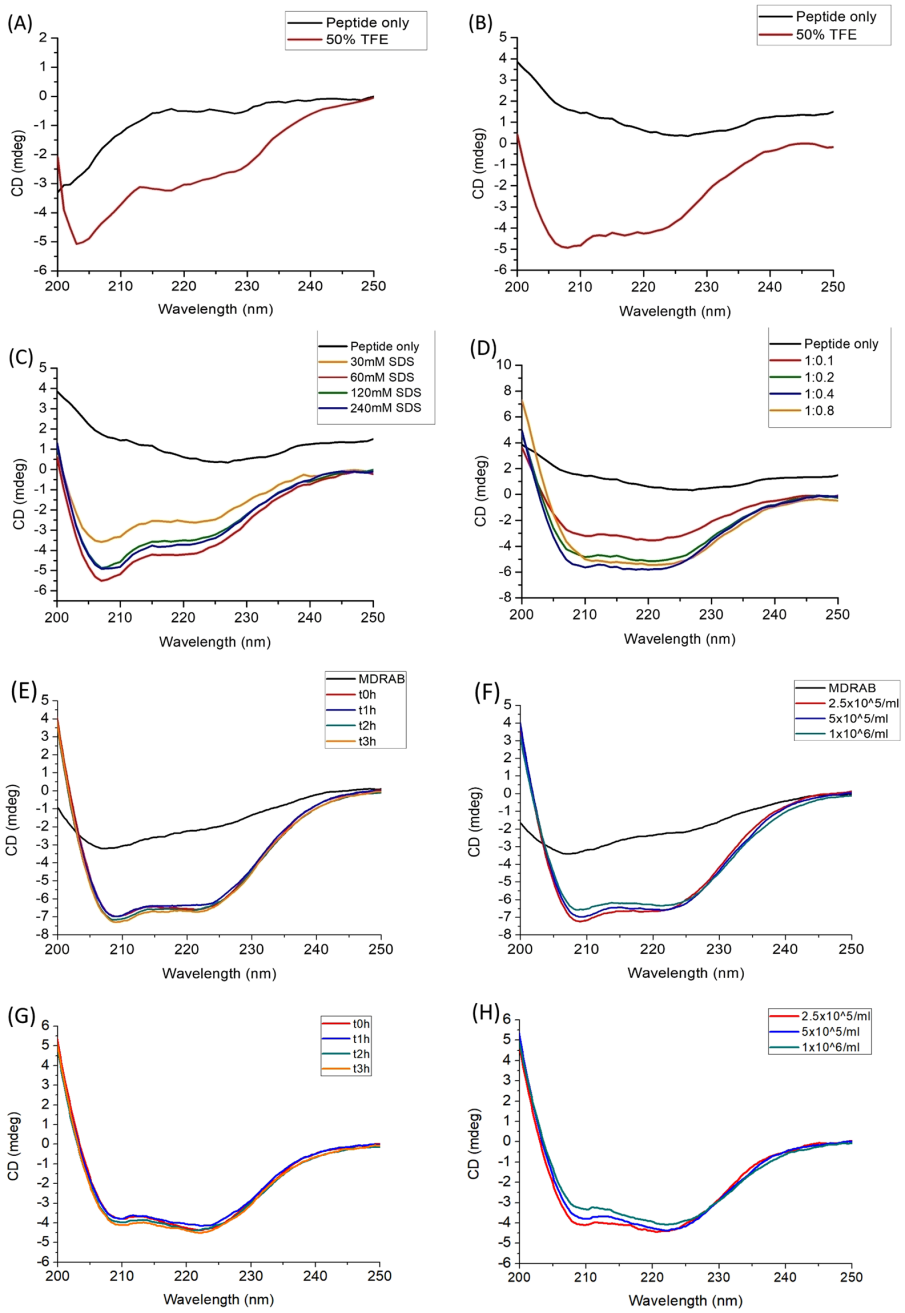
CD spectra of S1 wild type and SRP-2. (**A**) CD spectra of S1 wild type in PBS and 50% TFE. (**B**) CD spectra of SRP-2 in PBS and 50% TFE. (**C**) CD spectra of SRP-2 in different concentrations of SDS. (**D**) CD spectra of SRP-2 at different peptide:LPS molar ratios. The peptide concentration used in the experiments was 15 μM. (**E**) CD spectra of SRP-2 interacting with 5 × 10^5^ CFU/mL of MDRAB at different time (0, 1, 2, and 3 h). The black line is the CD spectrum of MDRAB sample without peptide. (**F**) CD spectra of SRP-2 interacting with different concentrations of MDRAB. 0 h spectra for different bacterial concentrations (2.5 × 10^5^, 5 × 10^5^, and 1 × 10^6^ CFU/mL) are presented. Black line is the CD spectrum of peptide free MDRAB sample at the concentration of 1 × 10^6^ CFU/mL. The bacteria controls in (**E**,**F**) are used for comparison. (**G**) CD spectra of SRP-2 interacting with 5 × 10^5^ CFU/mL of MDRAB at different time (0, 1, 2, and 3 h) after the spectrum of the bacterial cells was subtracted. (**H**) CD spectra of SRP-2 interacting with different concentrations of MDRAB in which the corresponding bacterial cell spectra were subtracted. 0 h spectra for different bacterial concentrations (2.5 × 10^5^, 5 × 10^5^, and 1 × 10^6^ CFU/mL) are presented.

From the AFM imaging process, we suggest that SRP-2 could interact and disrupt bacterial membranes to perform its bactericidal activity. Previous studies have indicated that the interactions of an AMP with membrane components should result in conformational changes of the peptide^44–46^; therefore, we applied CD spectroscopic measurements to examine the secondary structure changes of SRP-2 in membrane-mimicking environments. Moreover, 50% TFE has been used as one of the standard procedures for the induction of secondary structures of peptides in CD spectroscopy^47–50^. In CD measurements, we examined the structure of the S1 wild-type and SRP-2 peptides, and the CD spectra are illustrated in Fig. 5. Deconvolutions^37,51^ of the CD spectra were performed to calculate the related contents of the secondary structures of the peptides, and the results are shown in Supplementary Table S3. The wild-type peptide was in a typical random-coil conformation in PBS (Fig. 5A); the deconvolution of the spectra revealed that most of the structures were random coils and β-strands, which agreed with the secondary structure predictions. Upon interactions with 50% TFE, we observed α-helix spectral characteristics in the CD spectra; the α-helix content increased to 28% according to the deconvolution. According to the CD spectra, SRP-2 also adopted a random-coil and β-strand structure in PBS (Fig. 5B, Supplementary Table S3). In 50% TFE, clear and typical CD spectra indicating α-helical conformation were observed in the measurement of SRP-2 (Fig. 5B). We also explored the conformational changes of SRP-2 in membrane-mimicking environments. Here, we added SDS micelles into the solution to mimic the hydrophobicity of the biological membranes^52^ in order to evaluate possible effects on the peptide structures by peptide/biological membrane interactions. The CD spectra (Fig. 5C) revealed that SRP-2 in membrane-mimicking environments adopted typical and stable helical structures. Similar results were obtained in cases in which helical structures were induced when SRP-2 was treated with the bacterial membrane component LPS (Fig. 5D). The deconvolution of the CD spectra (Supplementary Table S3) indicated that α-helical structures of SRP-2 were indeed induced by the peptide interactions with TFE, SDS, and LPS and that SRP-2 should form α-helical structures upon interaction with biological membranes. As demonstrated by Avitabile *et al*.^36^, it is possible to measure CD spectra of peptide upon interactions with whole bacterial cells. Figure 5E,F shows our results of the CD spectroscopic measurements of SRP-2 interacting with MDRAB cells, while Fig. 5G,H shows CD spectra of SRP-2 when interacting with MDRAB cells, after the spectra of bacterial cells were subtracted. According to Fig. 5E,G, almost immediately, the peptide formed into α-helical structure when it was mixed with MDRAB at the concentration of 5 × 10^5^ CFU/mL. Decrease (to 2.5 × 10^5^ CFU/mL) or increase (to 2.5 × 10^5^ CFU/mL) of the bacterial concentration did not greatly change the α-helical contents of the peptide (Fig. 5F,H, and Supplementary Table S3). The results of CD spectroscopic measurements on peptide-bacteria interactions indicated that the association of SRP-2 to MDRAB is fast and strong.

### LPS neutralization

**Figure 6:**
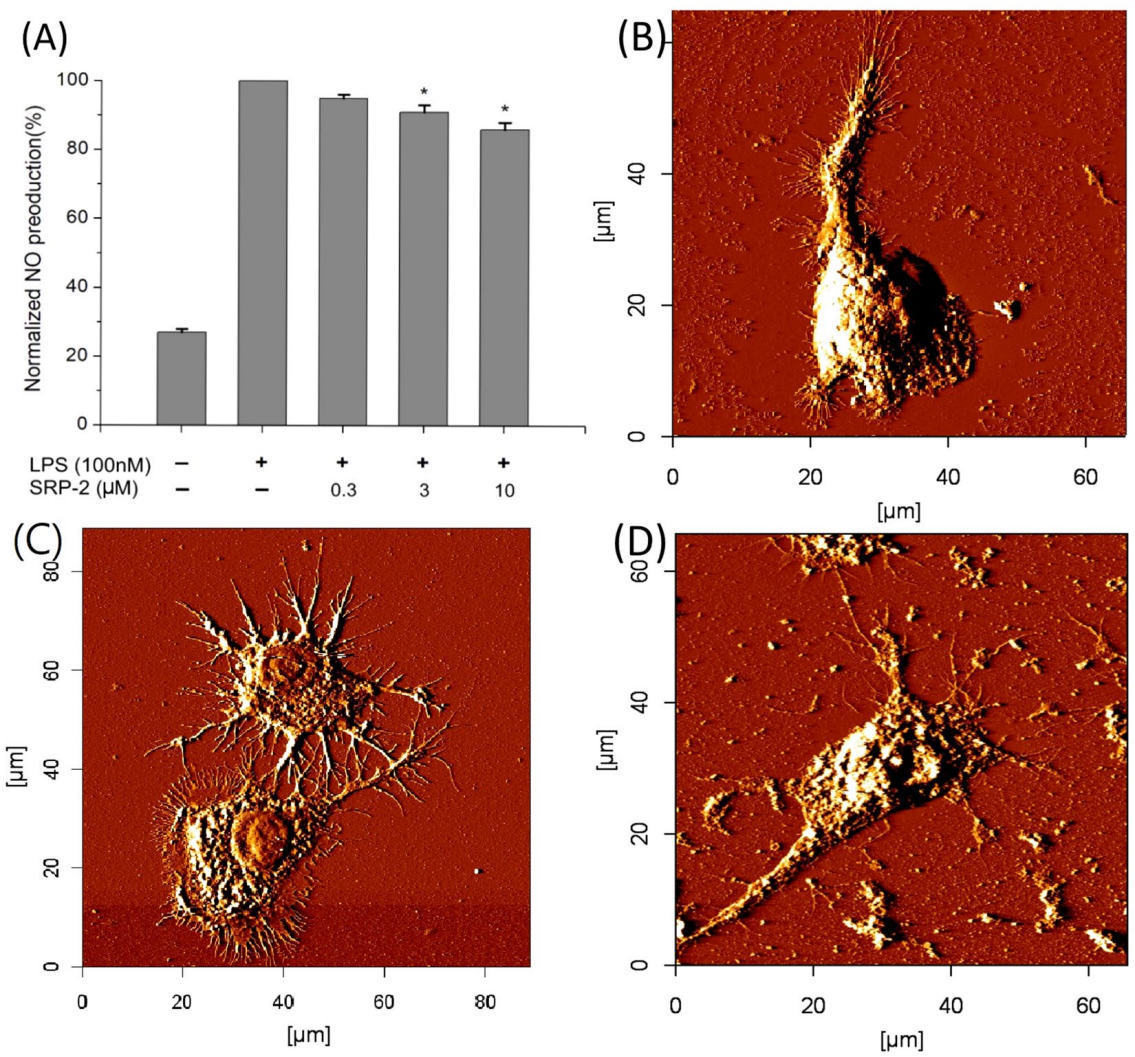
Attenuation of LPS-induced NO production and morphological changes in RAW 264.7 macrophages by SRP-2. (**A**) NO production measurements of RAW 264.7 cells cotreated with SRP-2 (0.3–10 µM) and LPS (100 ng/mL) for 24 h. Untreated cells served as the negative control, and LPS-treated cells served as the positive control. Statistically significant reductions in cellular NO production were measured in the groups treated with the peptide at concentrations of 3 and 10 μM. Values are mean ± SD. *p < 0.05, compared to the positive control group. (**B**) AFM image of an untreated RAW 264.7 cell. (**C**) Morphology of LPS (100 ng/mL)-activated RAW 264.7 cells, as imaged through AFM. As shown in the image, morphological changes and an increase in the number of extended pseudopodia on the cells were observed in LPS-activated cells. (**D**) Morphology of a RAW 264.7 cell cotreated with LPS (100 ng/mL) and SRP-2 (20 μM). According to the AFM images, SRP-2 treatments did indeed inhibit the LPS-induced morphological changes in RAW 264.7 cells.

LPSs, substances existing on the surfaces of Gram-negative bacterial cells and considered as bacterial endotoxins, are widely used in experiments for induction of inflammation *in vivo* and *in vitro*^53–55^. Because LPSs are negatively charged molecules, it is possible to use cationic amphipathic peptides to neutralize them^56^, thus reducing LPS-induced inflammation. In this study, we demonstrated the possible LPS neutralization effects induced by SRP-2 at cellular levels. LPSs can induce the activity of inducible NO synthase (iNOS) in RAW 264.7 macrophages. NO production, mainly catalyzed by iNOS, in macrophages is one of the indicators of the degree of inflammation^57^. In the present study, we examined the LPS neutralization effects on NO production by cotreating RAW 264.7 cells with LPS (100 ng/mL) and SRP-2 (0.3–10 μM). According to NO measurement results (Fig. 6A), although not strong, there was a dose-dependent reduction in NO production in RAW 264.7 cells with increased concentrations of SRP-2 in the treatments. However, the inhibitory effects on the LPS-induced morphological changes on RAW 264.7 cells by SRP-2 were more obvious, as visualized by AFM (Fig. 6B–D). Upon activation by LPS, an increase in the number of extended pseudopodia on the cells was observed by AFM (Fig. 6C) compared with the control group (Fig. 6B). When the cells were cotreated with LPS and SRP-2, the LPS-induced extension of pseudopodia was inhibited (Fig. 6D).

### SRP-2 on MDRAB-infected mice

**Figure 7:**
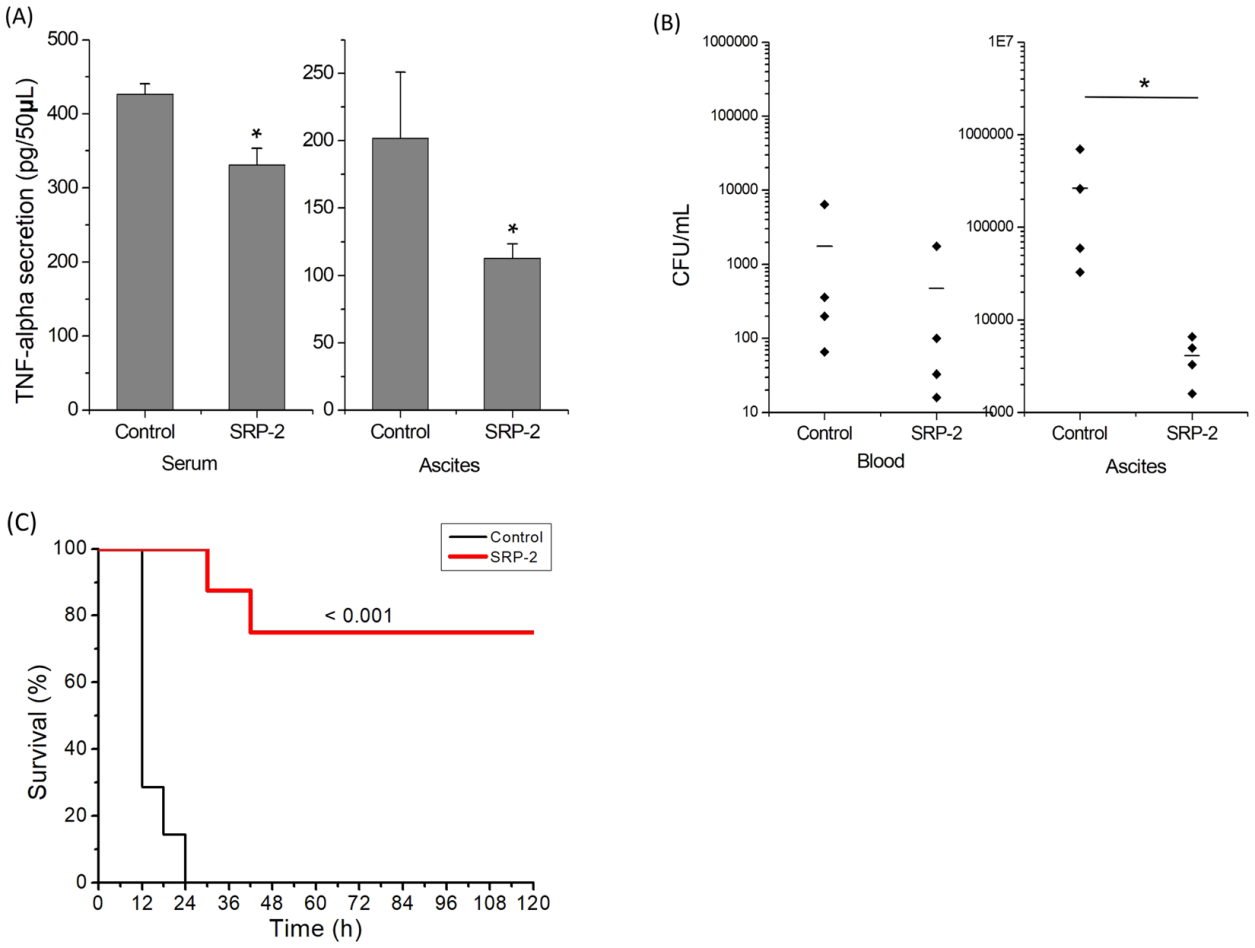
Bactericidal effects and inhibition of TNF-α secretion by SRP-2 in MDRAB-infected mice. (**A**) TNF-α secretion levels in serum and ascitic fluid of infected mice treated or untreated with SRP-2. Values are mean ± SD. *p < 0.05, compared to the control groups. (**B**) Bacterial counts in blood and ascitic fluid of infected mice treated or untreated with SRP-2. For the experiments, MDRAB (1 × 10^7^) were used to infect the mice. SRP-2 treatments were conducted through i.p. injection of the peptide (5 mg/kg mouse) to the infection sites 5 min after infection. Bacterial cell counts were determined 4 h after peptide treatments. *p < 0.05, SRP-2 treated group compared to the control group. (**C**) The survival of mice treated with a single dose of SRP-2 (10 mg/kg mouse) or PBS buffer i.p. 1 h after MDRAB infection (2 × 10^8^ CFU/mouse). The survival of the mice was tracked for 5 days.

We examined the effects of SRP-2 *in vivo*. BALB/c mice (8–10 weeks of age) were infected with MDRAB by i.p. injection, and 5 min later, the infected mice were injected with a single dose of SRP-2 at 5 mg/kg. The mice were euthanized 4 h later; TNF-α was measured, and viable bacteria were counted in the peripheral blood and ascitic fluid of the mice. The results are presented in Fig. 7. Significantly reduced TNF-α levels were measured in both the serum and ascites of infected mice when the mice were treated with SRP-2 (Fig. 7A). In the cases of viable bacterial levels (Fig. 7B), the numbers of bacteria in the blood were much lower than those in the ascitic fluid, which was from the major infection sites. According to the *in vivo* investigations, SRP-2 significantly reduced the number of viable bacteria at the infection sites and reduced the inflammation level in infected mice. As bacteremia is one of the common outcomes of *A*. *baumannii* infections, the ability of SRP-2 to rescue mice from lethal MDRAB infections was also investigated. Each animal received 2 × 10^8^ CFU of MDRAB through i.p. injection, and was treated 1 h later with a single dose of 10 mg/kg mouse of SRP-2 (n = 8) or PBS (n = 7) by the same route. While all of the PBS-treated mice died within 24 h after the infections, 75% (6 out of 8) of the SRP-2-treated mice survived from this highly lethal dose of MDRAB infection (Fig. 7C). The surviving mice started to behave normally from 48 h post-infection. These results collectively indicate that SRP-2 indeed exhibit great *in vivo* antimicrobial activity.

## Discussion

In this study, we designed three α-helical AMPs—namely SRP-1, SRP-2, and SRP-3—by performing amino acid substitutions in the template sequence of an S1 peptide from a horseshoe crab^28^ to increase the positive charges, helicity, and amphipathicity of the peptides, with the objective of yielding AMPs for broad-spectrum activities against both Gram-positive and Gram-negative pathogenic bacteria. Most of the effective AMPs contain net positive charges between +3 and +9^58,59^. Accordingly, we increased the peptide net positive charges to +8 in our design and reduced the numbers of negatively charged amino acids. Previous studies have suggested that a perfect amphipathicity of an α-helical peptide reduces the selectivity of the peptide and leads to severe damage to mammalian cells^60^. Therefore, in the design, we intentionally broke the perfect amphipathicity by inserting hydrophobic amino acids into the hydrophilic side of the helix (Fig. 1). The helical wheel projections revealed that the three designed peptides had similar distributions of cationic amino acids, in addition to having similar distributions of polar and nonpolar amino acids. However, the behaviors of these peptides in terms of broad-spectrum bactericidal activity and selectivity differed. The major differences were the cationic amino acids used. In SRP-3, only lysine was used to provide its positive charges. The experiments indicated SRP-3 to be effective against Gram-positive but not Gram-negative bacteria. Furthermore, this peptide caused severe hemolysis, rendering it unsuitable for applications inside an animal body. When three of the lysine residues on the hydrophilic side of the helix were replaced by arginine (SRP-1), the bactericidal activity against Gram-positive bacteria improved and the hemolysis caused was greatly reduced. The most notable finding was that when all the cationic residues in the peptide sequence were arginine, the peptide exhibited the greatest bactericidal activity against both Gram-positive and Gram-negative bacteria, and it did not induce hemolysis in the tests. AMPs rely on their positive charges to selectively interact with negatively charged bacterial membranes. Lysine and arginine carry equivalent positive charges under most physiological conditions; thus, they have been suggested to have similar chemical properties and play similar roles in proteins. However, our results show that they function differently in AMPs. The use of arginine results in superior bactericidal activity and selectivity compared with the use of lysine. Indeed, simulation studies have suggested that lysine might be deprotonated when in the membrane, whereas arginine would maintain its positive charge^61^. Furthermore, the guanidinium group of arginine allows interactions in three possible directions, enabling arginine to form a higher number of electrostatic interactions^62^. The higher pK_a_ of the arginine guanidinium group may also generate more stable ionic interactions than those generated by the amino group of lysine^62^. These might explain why arginine is a better choice than lysine for AMP design. A previous study on an arginine–lysine positional swap of AMPs derived from human cathelicidin LL-37 indicated the importance of the positions of arginine residues in AMP bactericidal activity and bacterial/mammalian cell selectivity^63^. Additionally, research on human AMP defensins revealed that the high arginine/lysine ratio of α-defensin-1 is essential for its bactericidal activity, and the mutant β-defensin-1 with its lysine residues replaced by arginine was functionally better than the original peptide^64^. Activity attenuation in mouse defensin cryptdin 4 by arginine-to-lysine substitutions was also documented^65^. These reports again indicate that arginine is superior to lysine for providing positive charges for AMP functions. In this study, SRP-2 was the only designed peptide with broad-spectrum activity, and it exhibited potent bactericidal activity against pathogenic Gram-positive MRSA and Gram-negative MDRAB. This peptide showed little effect on mammalian red blood cells or microvascular endothelial cells, HMEC1; thus, it can be considered safe for *in vivo* applications. Previous studies have suggested that many AMPs also exhibited anticancer activity^42,43^. However, despite its broad-spectrum activity against bacteria, SRP-2 did not seem to efficiently kill HepG2 cells.

Because an atomic force microscope can achieve imaging at extremely high resolutions on bacterial samples, it has recently become one of the popular tools for detailed imaging of bacterial cell structure and morphology^66–68^. Although AMPs can kill bacterial cells by adopting several strategies, including membrane disruption and functional inhibitions^16^, according to our AFM data, SRP-2 seems to interact with the cell membrane and cause direct bacterial cell disruption. The disruption of bacterial cell membrane by the peptide was further validated by fluorescence experiments using propidium iodide and flow cytometry (Supplementary Fig. S2).

In this study, the CD spectra of SRP-2 upon interactions with bacterial cells were also measured. It is indicated by the CD spectra that similar to those measured in secondary structure inducing agent (50% TFE), in membrane mimicking environment (SDS), or with LPS, the peptide is largely in α-helical structures when interacting with actual bacterial cells. According to the deconvolution results of CD spectra (Supplementary Table S3), the secondary structure contents of SRP-2 interacting with bacterial cells (based on the bacterial contribution subtracted peptide spectra) are close to those in 30 mM SDS, and those when the peptide interacts with LPS at the peptide:LPS molar ratio of 1:0.1. It is also worth noting that the α-helical structure of SRP-2 appear almost immediately when the peptide was mixed with the bacteria, and after 3 h of interaction with bacterial cells, SRP-2 still maintained its α-helical structure. This might imply that the interactions of the peptide with MDRAB cellular components are fast and strong, and the peptide is considerably stable when interacting with the bacteria. MDRAB might not have the ability to degrade SRP-2.

LPSs bind the receptors of many cells including monocytes, dendritic cells, and macrophages, and this binding promotes the secretion of NO and proinflammatory cytokines such as TNF-α^69,70^. In this study, we used positively charged SRP-2 to neutralize a negatively charged LPS, with the objective of inhibiting the LPS-induced effects on RAW 264.7 macrophages. In the case of LPS-induced NO reduction, the cotreatment of SRP-2 at 10 μM reduced NO production by approximately 10% in LPS-treated cells. However, the peptide effects on the LPS-induced morphological changes of the cells were more obvious as observed in AFM imaging. Upon activation by LPS, RAW 264.7 macrophage cells can change their morphology and form accelerated spreading and thus pseudopodia^71,72^. These cell morphological changes were visualized in our AFM imaging process (Fig. 6C). The cotreatment of SRP-2 on the LPS-treated cells greatly reduced the level of cell spreading and pseudopodia formations (Fig. 6D), indicating that SRP-2 can neutralize LPS in treatments. The effects of the SRP-2 treatments were also tested *in vivo* in a mouse model. Gram-negative MDRAB were injected into the peritoneum of mice for infections and to induce inflammation. As the bacteria were injected into the body cavity of the mice, the bacterial level in the peripheral blood was low; the peptide treatments reduced the bacterial number, but the reduction was not statistically significant. By contrast, the SRP-2 treatments did significantly reduce the number of bacteria in the ascitic fluid by approximately two orders of magnitude, indicating SRP-2 to be effective at killing bacteria in animal bodies. Infections of Gram-negative LPS-containing MDRAB results in the release of TNF-α^73,74^. According to our *in vivo* study, the TNF-α levels in infected mice were significantly reduced (in both blood and ascitic fluid) by the application of SRP-2. Moreover, a single dose treatment of SRP-2 through i.p. injection was able to rescue 75% of the mice infected with lethal dose of MDRAB.

The findings of this study demonstrate that bioinformatic calculation–assisted peptide design can be a powerful tool for the development of novel and potent AMPs. The peptide secondary structure predictions provided by the neural network–based bioinformatic prediction tool agreed adequately with the CD spectroscopic measurements. The designed arginine-rich SRP-2 can not only perform direct bacterial killing but also neutralize LPS in inflammation induction in both *in vitro* and *in vivo* experiments. We also found that arginine is better than lysine in the creation of positively charged AMPs with broad-spectrum bactericidal activity and selectivity of bacterial cells over mammalian cells. In addition to the potent AMP yielded in this study, we demonstrated the methodology for combining bioinformatic calculation and *in vitro*/*in vivo* experiments for the engineering and evaluation of AMPs. This methodology should also benefit further development of antibacterial agents.

## Electronic supplementary material

Supplementary Information
