# Supplementary material for: An engineered arginine-rich α-helical antimicrobial peptide exhibits broad-spectrum bactericidal activity against pathogenic bacteria and reduces bacterial infections in mice
Source: Sci Rep. 2018 Oct 2;8:14602. doi: 10.1038/s41598-018-32981-3 (PMC6168480; doi:10.1038/s41598-018-32981-3)
Supplement: Supplementary file 1 — Supplementary Information [file 41598_2018_32981_MOESM1_ESM.pdf]

An engineered arginine-rich α-helical antimicrobial peptide exhibits broad-spectrum bactericidal activity against pathogenic bacteria and reduces bacterial infections in mice

Chin-Hao Yang[1], Yi-Cheng Chen[2], Shih-Yi Peng[3], Andy Po-Yi Tsai[4], Tony Jer-Fu Lee[5,6], Jui-Hung Yen[7] & Je-Wen Liou[1,3]*

[1]Institute of Medical Sciences, Tzu Chi University, Haulien, Taiwan. [2]Department of Medicine, Mackay University, New Taipei, Taiwan. [3]Department of Biochemistry, School of Medicine, Tzu Chi University, Taiwan. [4]Ph.D. Program in Translational Medicine, Tzu Chi University/Academia Sinica, Taipei, Taiwan. [5]Department of Medical Research, Buddhist Tzu Chi General Hospital, Hualien, Taiwan. [6]Department of Pharmacology, Southern Illinois University School of Medicine, Springfield, IL, USA. [7]Department of Molecular Biology and Human Genetics, Tzu Chi University, Hualien, Taiwan. *Correspondence and requests for materials should be addressed to J.-W. L. (email: jwliou@mail.tcu.edu.tw)

Table S1. Predicted physical characteristic parameters of wild-type and designed peptides.

**Peptide**	**Net charge**	**Alpha helix %**	**Extended strand%**	**Random coil%**	μ**H**[1]	**H**[2]
**Wild type**	+4	0	35.29	64.71	0.258	0.491
**SRP-1**	+8	76.47	0	23.53	0.402	0.413
**SRP-2**	+8	88.24	0	11.76	0.403	0.498
**SRP-3**	+8	85.29	0	14.71	0.395	0.381

1. μH: mean amphipathic moment.

2. H: mean hydrophobicity.

Table S2. Minimum bactericidal concentrations (MBCs) of peptides against different bacteria.

Peptides	*E. coli*		*S. aureus*		MRSA		MDRAB	
	MBC (µM)		MBC (µM)		MBC (µM)		MBC (µM)	
	50%	90%	50%	90%	50%	90%	50%	90%
Wild type	>50	>50	>50	>50	NA	NA	NA	NA
SRP-1	>50	>50	6.25	12.5	NA	NA	NA	NA
SRP-2	<6.25	<6.25	<6.25	<6.25	0.5	1	0.5	1
SRP-3	>50	>50	12.5	25	NA	NA	NA	NA

Table S3. Secondary structure deconvolutions of peptide CD spectra.

**Peptide**	**Alpha helix %**	**Extended strand %**	**Random coil %**
**Wild type**			
PBS	7	51	42
50 % TFE	28	30	42
**SRP-2**			
PBS	3	50	47
50 % TFE	84	0	16
SDS 30 mM	56	10	33
SDS 60mM	86	0	14
Peptide:LPS<br>1:0.1	67	4	29
1:0.2	94	0	6
With MDRAB*<br>2.5 x 10^5 CFU/mL	61	9	30
5 x 10^5 CFU/mL	58	12	30
1 x 10^6 CFU/mL	56	13	31

*The secondary structure contents of SRP-2 when interacting with bacterial cells were calculated using the peptide spectra in which the bacterial cell spectra have been subtracted. The peptide spectra used were those taken immediately after the interactions (0 h).

Fig. S1

(A)

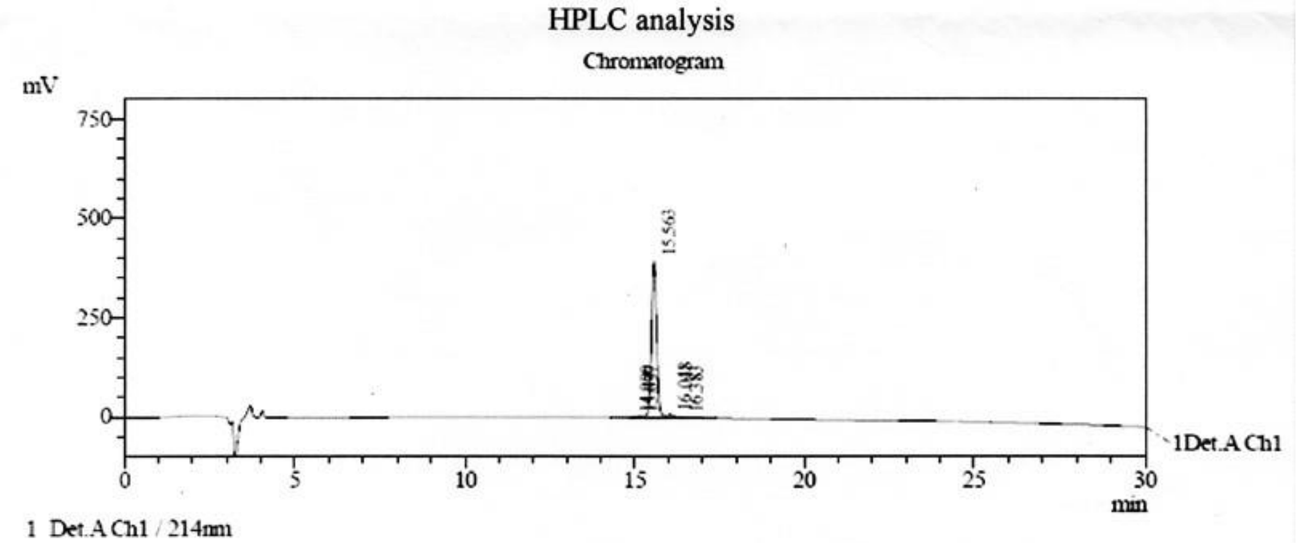

PeakTable

Detector A Ch1 214nm

Peak#	Ret. Time	Area	Height	Area %
1	14.900	20095	1663	0.452
2	15.037	37754	3087	0.849
3	15.563	4231415	395788	95.141
4	16.048	119610	7664	2.689
5	16.383	38648	1530	0.869
Total		4447522	409731	100.000

(B)

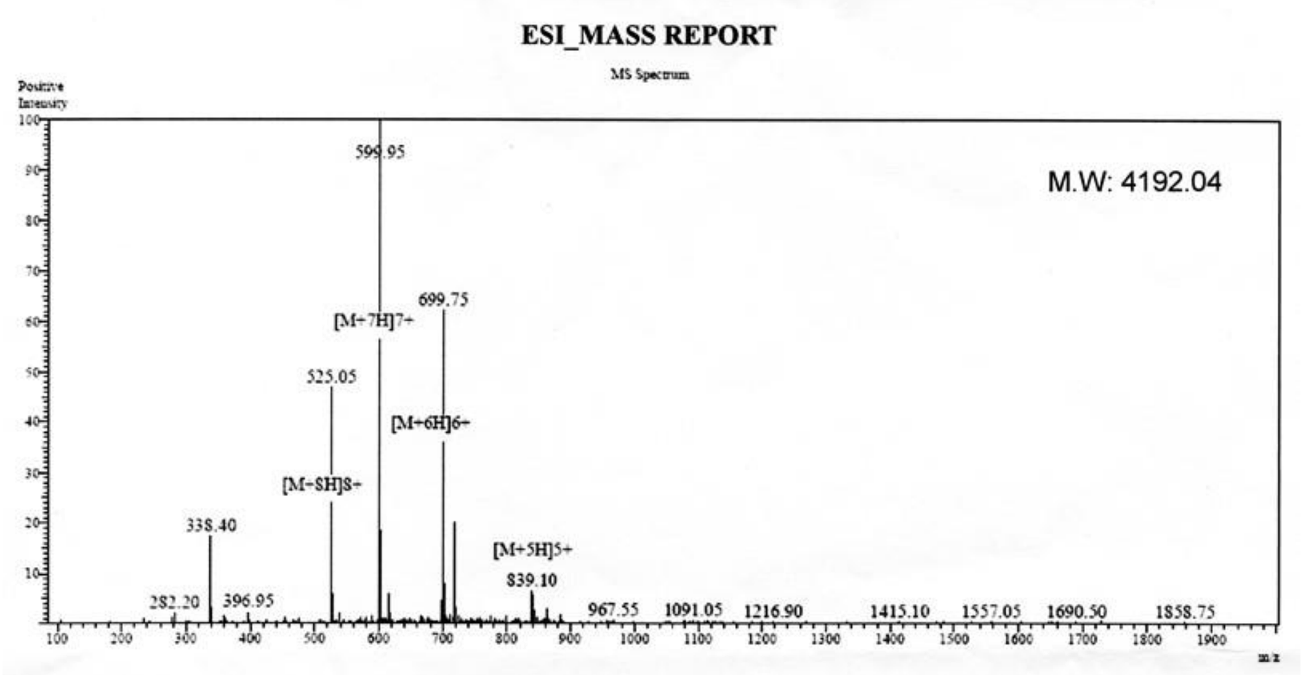

(C)

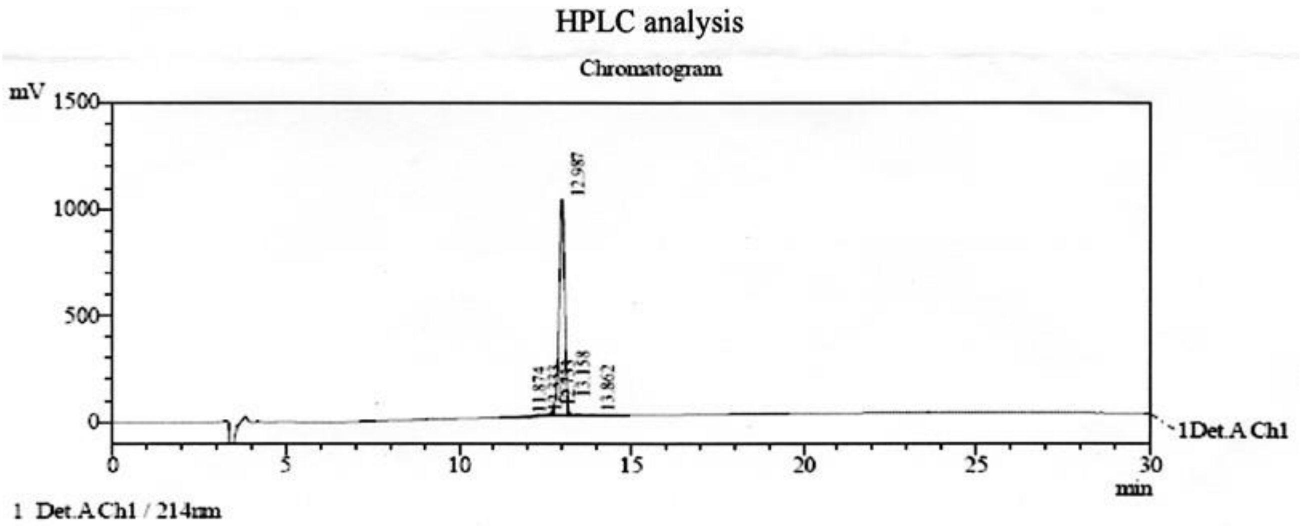

PeakTable

Detector A Ch1 214nm

Peak#	Ret. Time	Area	Height	Area %
1	11.874	13267	1918	0.114
2	12.333	110204	7176	0.951
3	12.733	143580	31253	1.239
4	12.987	11066969	1018953	95.474
5	13.158	232925	73956	2.009
6	13.862	24712	3495	0.213
Total		11591656	1136750	100.000

(D)

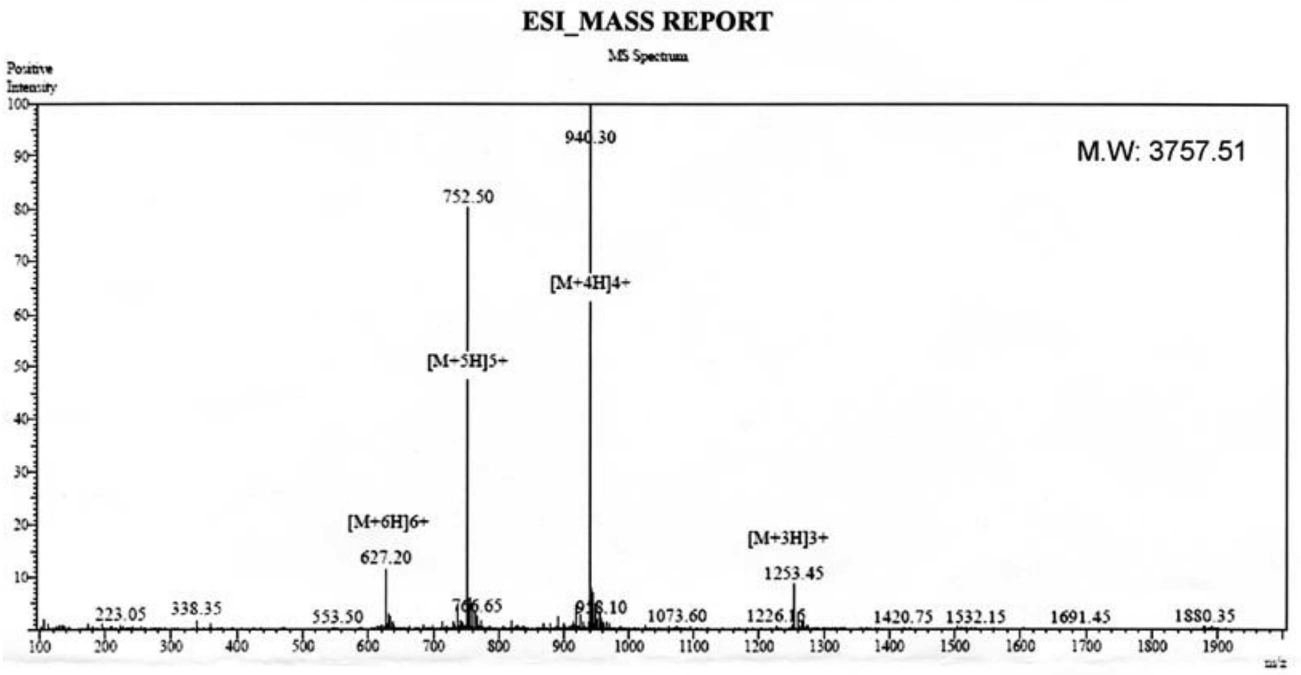

Figure S1. Representative results of peptide purity checks. The purified peptides were purity checked with HPLC and ESI mass spectrometry. (A) is a HPLC analysis chromatogram of purified SRP-2, the purity of the peptide is 95% according to the area percentage of the peak. (B) is the identity confimation of the SRP-2 peak in (A) using mass spectrometry. (C) is a HPLC analysis chromatogram of purified S1 wild type peptide, the purity of the peptide is 95% according to the area percentage of the peak. (D) is the identity confimation of the S1 wild type peptide peak in (C) using mass spectrometry.

Fig. S2

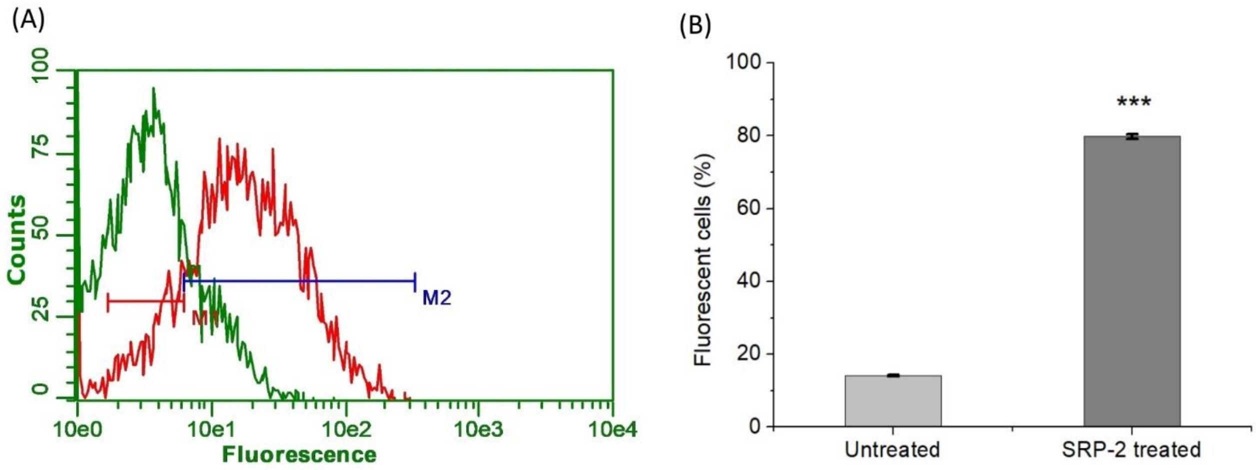

Figure S2. Flow cytometric analysis of SRP-2 treated MDRAB cells stained with propidium iodide (PI). (A) The fluorescence distributions of PI stained untreated (green) and SRP-2 treated (red) bacterial cells. (B) The percentages of fluorescent bacterial cells (M2 %) in PI stained untreated and SRP-2 treated samples. Results represent mean ± SD from triplicate experiments, ***p<0.001, compared to the untreated group. For the measurements, mid-logarithmic phase bacterial cells at a concentration of 1 x 10^8 CFU/mL in PBS, pH7.4 were used for experiments. For peptide treated samples, bacteria were treated with SRP-2 at a final concentration of 5 µM for 1 h at 25ºC. 1 mL of the bacterial samples (treated or untreated with SRP-2) were added with 5 µL of 50 mg/mL PI solution. After 10 minutes incubation at 25ºC, the cell samples were analyzed on a flow cytometer (Guava EasyCyte Mini, Guava Technologies/Millipore, USA). The elevated fluorescence in bacterial cells indicates the increase in membrane permeability resulting from the disruption of the cells by SRP-2 treatments.
